# Development of myelofibrosis and acute monocytic leukemia in a patient with hereditary spherocytosis: A case report

**DOI:** 10.1097/MD.0000000000018266

**Published:** 2019-12-16

**Authors:** Juan Qian, Qian Shen, Hong Yin, Wen-yu Shi, Li Yang, Ya-ping Zhang, Hong Liu

**Affiliations:** a Department of Hematology, Affiliated Hospital of Nantong University; b Department of Oncology, Nantong Oncology Hospital, Nantong, Jiangsu, China.

**Keywords:** acute monocytic leukemia, hereditary spherocytosis, myelofibrosis

## Abstract

**Introduction:**

Common symptoms of hereditary spherocytosis (HS) include intermittent jaundice and splenomegaly. Here, we present an unusual clinical course wherein a patient with HS treated with splenectomy developed secondary myelofibrosis and acute monocytic leukemia (M5).

**Patient concerns:**

After presenting with paleness, fatigue and jaundice, the patient was diagnosed with HS. After splenectomy, follow-up testing, including bone marrow biopsy, revealed myelofibrosis. Subsequently, the patient exhibited blood cell abnormalities consistent with M5.

**Diagnosis:**

M5 comorbid with myelofibrosis and a history of HS.

**Interventions:**

HS was treated with splenectomy. Myelofibrosis was treated with hydroxyurea. The patient refused chemotherapy for M5 and was discharged. He was maintained on hydroxyurea and received periodic blood product transfusions with regular routine blood test monitoring.

**Outcomes:**

Because of intracranial hemorrhage, the patient died on May 17, 2018, a little >10 months after being diagnosed with leukemia.

**Conclusion:**

The present patient developed M5 while undergoing treatment for myelofibrosis and after undergoing splenectomy for HS, raising the question of whether these conditions might be associated. Examination of this question will require the analysis of additional cases.

**Core tip**

Several recent case reports have described hematological malignancies deriving from HS, including chronic myelogenous leukemia and acute lymphoblastic leukemia. HS can present at any age from infancy to old age, with signs and symptoms due to hemolytic anemia or its complications. However, M5 is not an established derivative comorbidity of HS.

## 1 Introduction

Hereditary spherocytosis (HS) is a form of hemolytic anemia caused by congenital red blood cell (RBC) membrane defects. Patients with HS may present with intermittent jaundice and splenomegaly. HS can present at any age from infancy to old age, with signs and symptoms due to hemolytic anemia or its complications.^[1]^ Splenectomy can improve HS symptoms. The incidence rate of HS in northern Europe and North America approaches 1/2000.^[2]^ In northern China, some cases of HS have been reported in which spherocytosis was identified as the main cause of hereditary hemolytic anemia.^[3]^ Wang et al^[4]^ reported a case of HS that was associated with chronic myelocytic leukemia; Zhang et al^[1]^ described the case of a woman with comorbid HS and chronic myelocytic leukemia who underwent allogenic stem cell transplantation from a sibling donor. Kies et al^[5]^ described the case evolution of a patient with HS who suffered relapse associated with enlargement and leukemic myeloblast-infiltration of residual splenic tissue 8 years after a splenectomy was performed. Thus far, however, acute myeloid leukemia secondary to HS has been observed extremely rarely.

Here, we report a case of HS that converted to a subtype of acute myeloid leukemia known as acute monocytic leukemia (M5) with long-term extant bone marrow (BM) fibrosis. To our knowledge, a case with this clinical progression has never been reported. Context for this novel case is provided with a brief review of the relevant literature.

## 2 Case presentation

A 50-year-old male presented at our hospital in August 2009 with complaints of being pale and fatigued over the previous 3 months. In a physical examination, the spleen was palpated 2 cm below the left costal margin; the liver was not detected. Mild yellowish discoloration of the skin and sclera was noted. Routine blood and liver-function tests were performed (Table 1). An erythrocyte osmotic fragility test yielded abnormal findings: hemolysis began and ended at 0.60% and 0.40% salt concentrations, respectively (normal ranges. 0.44%–0.48% and 0.28%–0.36%, respectively; Chinese Academy of Medical Sciences Blood Disease Hospital).

**Table 1:**
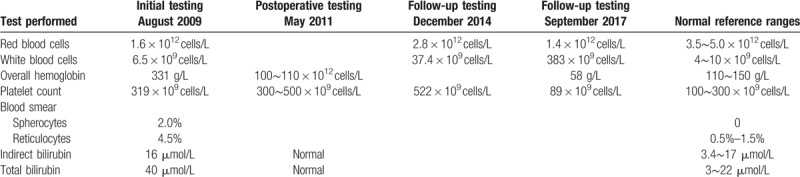
Routine blood test results.

**Figure 1:**
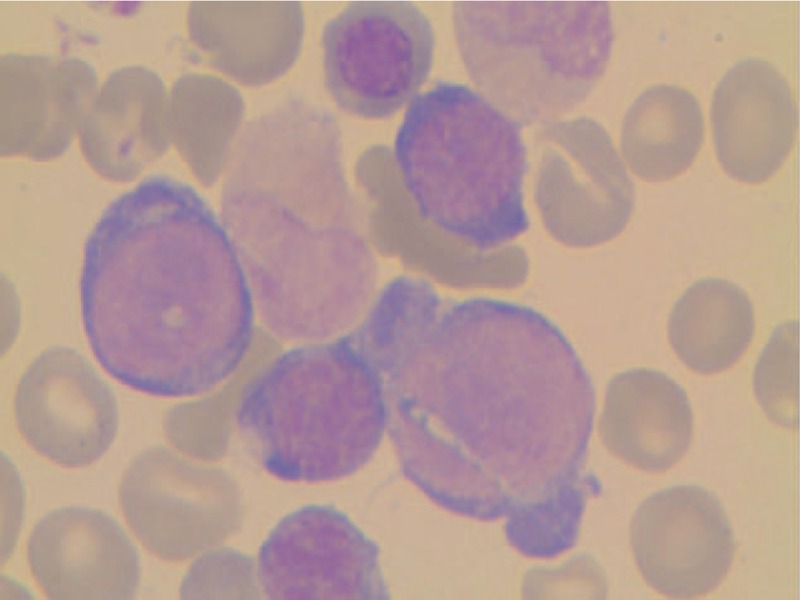
Bone marrow (BM) test in August 2009 shows hyperactive BM cell proliferation as well as RBC proliferation (Wright staining, 1000× magnification).

Routine BM tests showed active proliferation of RBCs and BM cells (Fig. 1), with the former exhibiting hyperactivity. Both the size and color of the mature RBCs present were atypical, with the latter being partly polychromatic. RBC debris was also observed together with intact spherical RBCs. Chromatin bodies were present in the cytoplasm. Folic acid, vitamin B12, ferritin, and glucose-6-phosphate dehydrogenase test results were normal. Coomb test showed a negative result. Hemoglobin exhibited normal electrophoresis, and the expression levels of CD59 and CD55 on granulocytes and RBCs were within normal range. A genetic analysis detected a V617 mutation in *JAK2* with a normal karyotype (46XY). The patient's daughters, brothers, and sisters had positive RBC osmotic fragility test results indicative of anemia. All family members tested had a history of dizziness.

Based on these clinical, laboratory, biochemical, and genetic tests, a clinical diagnosis of HS was made. The patient was prescribed methylprednisolone (40 mg/day) and folate supplements (2.5 mg/days). His symptoms and signs were not improved 18 months later. The patient underwent a splenectomy in May 2011, which alleviated the yellowing of his skin and sclera. Routine blood tests (Table 1) indicated that the patient's hemoglobin and bilirubin levels had returned to normal, although his overall hemoglobin levels were reduced to one-third of normal levels.

**Figure 2:**
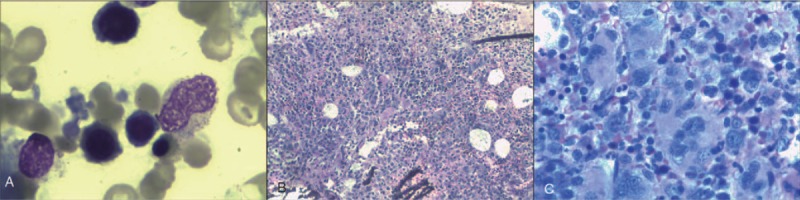
Follow-up tests performed in December 2014. (A) Routine bone marrow (BM) test showing active BM cell proliferation with megakaryocytes present. (B) Periodic-acid Schiff and hematoxylin-eosin staining showing hyperactivity of myelodysplastic cancer cells (90%) in BM biopsy sections. (C) Reticular fiber staining of BM demonstrating grade 2 marrow fibrosis.

Follow-up routine blood tests performed in December 2014 showed increased counts of white blood cells, RBCs, and platelets compared with the initial levels detected in August 2009 (Table 1). Peripheral blood smears showed a nucleated RBC ratio of 100:1, abnormal mature RBC size, and a subset of RBCs exhibiting a teardrop shape. The neutrophil alkaline phosphatase positivity rate was 79%, with a score of 266. BM tests showed active proliferation of BM cells and small megakaryocytes (Fig. 2A). Periodic-acid Schiff and hematoxylin-eosin staining of BM biopsy samples showed hyperactivity of myelodysplastic cells (90%) (Fig. 2B). The proportion of grain red was also abnormally high. Granulocytes exhibited a mild nuclear left shift, whereas RBCs included mainly middle and late young RBCs. Reticulin staining revealed grade 2 marrow fibrosis and megakaryocytosis with megakaryocytes in a scattered distribution mixed with small clusters (Fig. 2C). Based on these results, a clinical diagnosis of myelofibrosis was made, and continuous hydroxyurea was prescribed at a dose that was adjusted according to blood test results.

**Figure 3:**
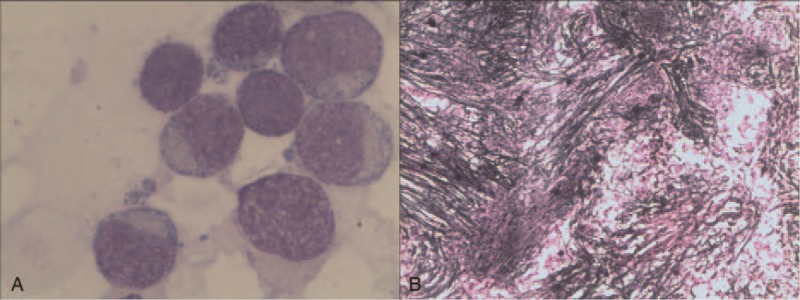
Follow-up tests performed in September 2017. (A) Routine bone marrow (BM) test showing marked BM cell proliferation, with primary and young mononuclear cells accounting for 85% of these active cells. Most of the active cells were peroxidase-negative. (B) BM biopsy sample with strong reticulin fiber staining.

In September 2017, routine blood examinations showed a 10-fold increase in white blood cell count, a 50% decrease in RBC count, and an approximately 5-fold decrease in platelet count (Table 1). Meanwhile, the patient's overall hemoglobin decreased to 58 g/L. BM tests revealed widespread active BM cell proliferation, with primary and young mononuclear cells accounting for 85% of the cell population examined. Furthermore, most of the cells were negative for peroxidase staining (Fig. 3A). A BM biopsy showed strong reticulin fiber staining (Fig. 3B). Accordingly, the patient received a clinical diagnosis of acute monocytic leukemia (M5) with myelofibrosis. Two-generation gene sequencing detected a D894G mutation in *SF3B1* (45.8%, 912/1993), an R550∗ mutation in *TET2* (44.5%, 884/1986), an R1465∗ mutation in *TET2* (50.2%, 1003/1999), a V617F mutation in *JAK2* (95.1%, 1898/1996), a Q778∗ mutation in *ASXL1* (49.9%, 996/1994), and an R1297∗ mutation in *BCORL1* (75.6%, 1178/1559) (Table 2).

**Table 2:**
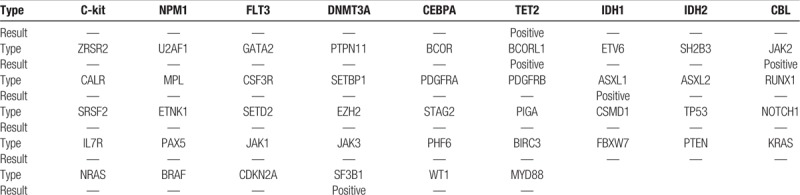
Two-generation gene sequencing was performed and the following mutations were detected: a D894G mutation in *SF3B1* (45.8%, 912/1993); a R550∗ mutation in *TET2* (44.5%, 884/1986), a R1465∗ mutation in *TET2*- (50.2%, 1003/1999); a V617F mutation in *JAK2* (95.1%, 1898/1996); a Q778∗ mutation in *ASXL1* (49.9%, 996/1994); and a R1297∗ mutation in *BCORL1* (75.6%, 1178/1559).

The patient refused recommended hospitalization and chemotherapy due to economic reasons and was therefore discharged. The patient was submitted to regular routine blood tests, continued to take hydroxyurea and accepted periodic transfusions of blood products. Because of intracranial hemorrhage, he died on July 27, 2018, a little >10 months after being diagnosed with leukemia.

## 3 Discussion

HS has been associated with heterogeneous modes of inheritance, various protein defects, and a spectrum of clinical severity.^[1]^ Correspondingly, clinical, laboratory, and genetic test results vary substantially among patients with HS.^[6,7]^ Although there is no consensus regarding hematological malignancy risk in HS patients, HS has been suggested to be a potential preleukemic state.^[8]^ Several recent cases of HS-derived hematological malignancies, including chronic myelogenous leukemia and acute lymphoblastic leukemia, have been reported. Very few cases of HS-derived acute myeloid leukemia have been reported. Notably, Kies et al^[5]^ reported the case of a white man with HS who exhibited hemolysis 8 years after undergoing a splenectomy due to a supernumerary spleen; 4 months later, acute myelomonocytic leukemia infiltration of the supernumerary spleen was diagnosed.

The present patient developed M5 while undergoing treatment for myelofibrosis, which had emerged 5 years after undergoing splenectomy for HS, leading us to wonder whether these conditions were associated. Second-generation sequencing revealed malignant clones. After being diagnosed with myelofibrosis, our patient exhibited several risk factors of leukemia, such as high white blood cell counts, high platelet counts, being >50 years’ old, and carrying a *JAK2* mutation. His disease course was consistent with the development of classical primary myelofibrosis.^[9]^

A report of acute promyelocytic leukemia complicated with myelofibrosis in the past.^[10]^ Given the absence of other reports describing this unique nexus of conditions, there is insufficient evidence to enable us to distinguish whether the present progression occurred by chance or as a consequence of pathogenic interactions. However, the prognosis of secondary acute myeloid leukemia after myeloproliferative tumor may be worse than that of primary acute myeloid leukemia.^[11]^ It will be of great interest to explore additional cases, as they are reported to determine whether associations exist among HS, myelofibrosis, and M5 (or acute myeloid leukemia in general).

## Author contributions

**Data curation:** Juan Qian, Qian Shen, Li Yang, Hong Liu.

**Formal analysis:** Juan Qian, Hong Yin, Hong Liu.

**Funding acquisition:** Juan Qian, Wenyu Shi, Li Yang, Yaping Zhang, Hong Liu.

**Investigation:** Juan Qian, Hong Liu.

**Methodology:** Qian Shen, Wenyu Shi, Li Yang.

**Project administration:** Hong Yin.

**Resources:** Hong Yin, Wenyu Shi.

**Software:** Yaping Zhang.

**Supervision:** Yaping Zhang.

## References

[1] Zhang XH Fu HX Xu LP Allo-hematopoietic stem cell transplantation is a potential treatment for a patient with a combined disorder of hereditary spherocytosis. Chin Med J (Engl) 2012;125:947–50. 22490603

[2] Bolton-Maggs PH Stevens RF Dodd NJ Guidelines for the diagnosis and management of hereditary spherocytosis. Br J Haematol 2004;126:455–74. 15287938 10.1111/j.1365-2141.2004.05052.x

[3] Zhang Bihong Chen Chun Cen Danyang 26 cases of hereditary spherocytosis. J Applied Clin Pediatrics 2008;23:1162–4.

[4] Wang X Fang B Jiang J A case report of hereditary spherocytosiswith concomitant chronic myelocytic leukemia. Open Med 2016;11:152–4. 10.1515/med-2016-0029 PMC5329816 28352784

[5] Kies MS Abbott OD Rubin RN Recurrence of hemolysis in hereditary spherocytosis: a case due to leukemic infiltration of an accessory spleen. Mil Med 1981;146:55–7. 6784002

[6] Kaysser TM Wandersee NJ Bronson RT Thrombosis and secondary hemochromatosis play major roles in the pathogenesis of jaundiced and spherocytic mice, murine models for hereditary spherocytosis. Blood 1997;90:4610–9. 9373273

[7] White B Leong KW Crotty GM Hereditary spherocytosis: implications in bone marrow transplantation. Bone Marrow Transplant 1998;21:215. 9489641 10.1038/sj.bmt.1701059

[8] Ishida Y Niino M Matsuda H Hereditary spherocytosis presenting with acute lymphoblastic leukemia. Rinsho Ketsueki 1987;28:402–7. 3475487

[9] Vannucchi AM Lasho TL Guglielmelli P Mutations and prognosis in primary myelofibrosis. Leukemia 2013;27:1861–9. 23619563 10.1038/leu.2013.119

[10] Abou Dalle I Nassif S Bazarbachi A Acute promyelocytic leukemia with increased bone marrow reticulin fibrosis: Description of three cases and review of the literature. Hematol Oncol Stem Cell Ther 2018;11:99–104. 27614232 10.1016/j.hemonc.2016.08.001

[11] Chiche E Bonnet S Betoli S Number of mutations and type of prior myeloproliferative neoplasm are prognostic factors in acute myeloid leukemia post myeloproliferative neoplasms. Blood 2018;132:2806.

